# Recurrent Parotid Carcinosarcoma in an Asymptomatic Patient

**DOI:** 10.1177/2324709616676590

**Published:** 2016-11-04

**Authors:** Joshua Mansour, Abhishek Mangaonkar, Vamsi Kota

**Affiliations:** 1 Medical University of South Carolina, Charleston, SC, USA; 2 Mayo Clinic, Rochester, MN, USA; 3 Emory University, Atlanta, GA, USA

**Keywords:** oncology, rare tumor, head and neck cancer, radiotherapy

## Abstract

In this article, we present the case of a 52-year-old male with a history of parotid carcinosarcoma with initial diagnosis being 18 months prior. Initial treatment included a combination of gamma knife surgery coupled with high dosage chemotherapy and X-ray radiation therapy. At the time of follow-up, the patient presented with no complaints and had a nearly normal physical exam with the exception of some facial nerve weakness on the same side as the initial surgery. Despite being asymptomatic, the patient had a significant progression of disease that was manifested with intracranial lesions, multiple pathologic fractures, and a dramatic increase in overall tumor burden. Ultimately, the patient decided to pursue comfort measures only and succumbed to the disease peacefully soon thereafter.

## Introduction

Carcinosarcoma is a rare salivary gland tumor that contains numerous different cell types. Moreover, these tumors are generally high grade, highly invasive, often metastatic, and frequently recurrent. Carcinosarcomas classically contain a bimodal cell population, which includes both epithelial and mesenchymal components that are superimposed on each other when viewed histologically.^1^ These tumors may be seen in different glands of the mouth, varying from the parotid gland to minor salivary glands.^2-4^ These tumors strike a very wide age range from 14 to 87 years but seem to have an average age of about 58 years at time of presentation^5^ (Table 1).

**Table 1.** Summary of Current Knowledge on Salivary Carcinosarcomas^1-14^.

Number of cases reported in published literature	<73 cases
Percentage of malignant salivary gland tumors that are carcinosarcoma	0.16% to 1.0%
Percentage of carcinosarcomas in the salivary gland that arise from parotid	65%
Percentage of carcinosarcomas that arise from the submandibular gland	22%
Average age at presentation	58 years
Gender predominance	None
Percentage that experience recurrence	66% to 67%
Percentage that experience metastasis	50% to 54%
Median period of survival after diagnosis	10 months in 63%
Mean survival time	2.5-3.6 years

## Case Presentation

The patient was 52-year-old Caucasian male, with no significant past medical history, who presented for routine follow-up after having been diagnosed with carcinosarcoma of the right parotid gland with a metastatic deposit within the jugular foramen, 18 months after diagnosis and 1 year after completion of treatment. He was an avid smoker of 1 to 1.5 packs per day, did not have a family history of cancer, and had no environmental exposures during work.

At the time of diagnosis, 18 months prior, initial pathological and histological analysis displayed a malignant neoplasm of the parotid gland with a biphasic histological pattern. The minority component had overtly glandular epithelial features characterized by well-defined islands of neoplastic cells. These cells were positive for pankeratin and epithelial membrane antigen. Special stains also revealed intraluminal/intracystic mucin. Surrounding the aforementioned neoplastic islands were cells showing a solid growth pattern. These cells were negative for pankeratin and only a small number showed staining for epithelial membrane antigen.^6,7^

After discussion and presentation at tumor board the patient had started an aggressive treatment regimen that included a radical parotidectomy, complete with neck dissection and excision of the jugular foramen mass using gamma knife radiosurgery. Given the aggressiveness of the disease, on completion the patient was referred to medical oncology for concurrent chemoradiation. At that time the patient received 3 cycles of cisplatin chemotherapy at a dosage of 100 mg/m^2^ along with X-ray radiation therapy. The positron emission tomography (PET) scan following completion of treatment showed no evidence of local recurrence within the parotid or mastoid region. Furthermore, there was no radiological evidence of distal recurrence using flurodeoxy glucose–labeled PET scanning.

At this time the patient had then presented to the clinic for a routine 6-month follow-up visit (a year after completion of therapy) with no complaints or symptoms. The oral cavity and oropharyngeal examination revealed no lesions on presentation. Both ear canals were patent and the patient’s tympanic membranes were clear. Visual acuity was unchanged and hearing was intact. Facial sensation was intact; however, the patient showed absent motor response on the right side, which had presented after initial therapy and was since unchanged. Palate elevation as well as shoulder strength was symmetric, the tongue was mobile bilaterally, and no other abnormalities were noted on a complete examination. The patient’s vital signs were stable and within normal limits with temperature of 36.1°C, blood pressure of 124/77 mm Hg, a pulse of 74, and a respiratory rate of 16.

**Figure 1.**
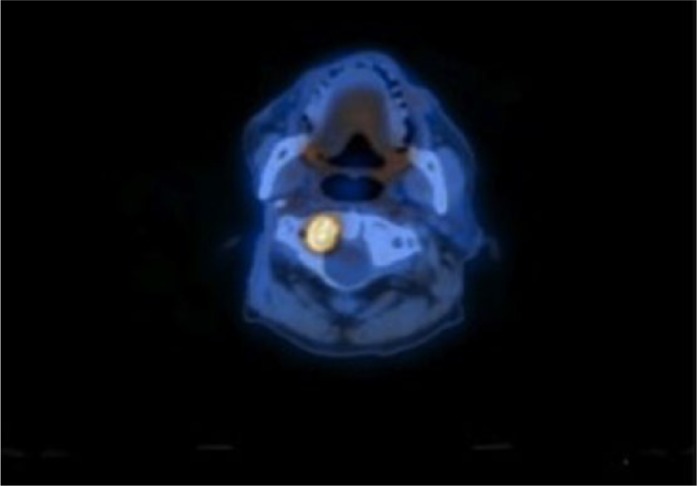
PET/CT scan shows the recurrence of the carcinosarcoma including a metastatic lesion that is in the C1 region.

**Figure 2.**
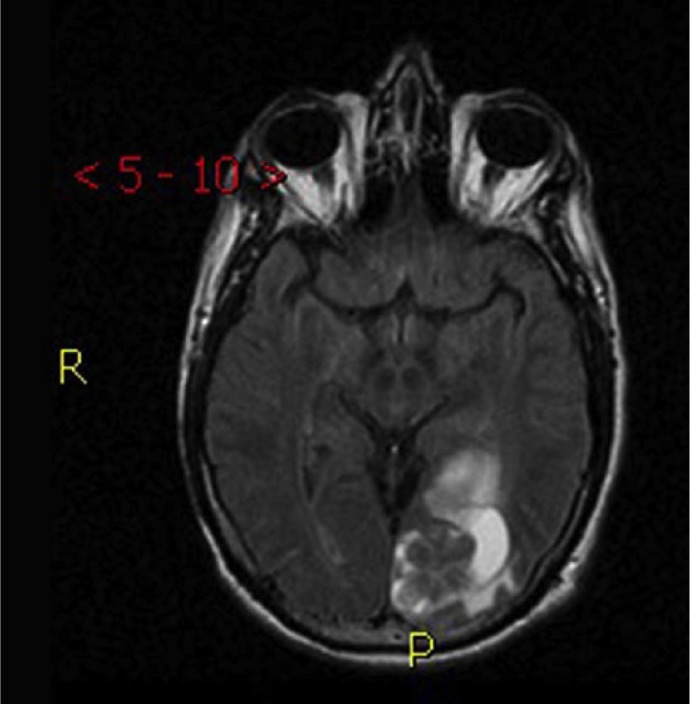
Magnetic resonance imaging scan shows the dramatic signal attenuation in the region of a large metastatic mass within the cranial vault coupled with resultant vasogenic edema.

Repeat PET and computed tomography (CT) scan were done following patient’s clinic visit, which had displayed widespread bone metastases. Multiple metastases in the spine were of concern regarding pathologic fracture potential, especially at C1 (as seen in Figure 1). There were new areas of hemorrhage in the brain with surrounding edema that were noted as well (as seen in Figure 2). Areas including the posterior left occipital lobe and posterior parasagittal left parietal lobe showed hemorrhagic metastases although the patient remained asymptomatic.

The magnetic resonance imaging of the brain also showed significant metastatic activity. There was a lobulated enhancing intra-axial mass lesion within the left precuneus that measures 2.7 cm × 1.6 cm × 1.8 cm, and a large enhancing lobulated mass lesion occupying a large portion of the left occipital lobe that measured approximately 2.5 cm × 4.1 cm × 3.8 cm. There was vasogenic edema around larger lesions located within the left precuneus and left occipital lobe with resultant mass effect on the atrium (as seen in Figure 2).

There has been marked progression of widespread bone metastases throughout the spinal column including but not limited to the following new metastases at C4, right lamina of T1 with lytic destruction extending into the spinal canal, lytic left anterior T4, including left fifth rib posterolaterally. Additional lytic metastasis were seen on the left section of T8 with a larger and a much more intense, right transverse process of T9, left T11, and at the right anterior region of T12. However, there was no recurrent tumor seen in the right parotid bed.

On repeat biopsy a well to moderately differentiated adenocarcinoma with strikingly cribriform growth pattern and large areas of necrosis was seen. The tumor morphology was similar to that of the carcinomatous portions of the previously diagnosed carcinosarcoma of parotid, indicating that the metastatic brain tumor likely represented a metastatic salivary ductal adenocarcinoma component of carcinosarcoma.

The cancer therefore recurred in this asymptomatic patient nearly a year since completion of aggressive treatment, which consisted of a combination of surgery, gamma knife surgery, chemotherapy, and radiation. Unfortunately, with the dramatic increase in tumor burden, as well as the patient not wanting further aggressive treatment, the patient opted for hospice care and peacefully succumbed to his disease.

## Discussion

In the present case, we present an asymptomatic patient with a recurrent metastatic carcinosarcoma. The known metastasis included to the ribs, spinal column, and brain. Carcinosarcoma is by definition a tumor with a bimodal cell population that is aggressive, and generally has high-grade cell morphology under histological analysis.^2,4^ In general, radical surgical exploration coupled with high dosage chemotherapy coupled with radiation are necessary for treatment. However, even with this aggressive regimen disease reoccurs in about 66% of patients, with metastatic dissemination in about 50%^8,9^ (Table 1). Interestingly, the lungs seem to be the most common site of dissemination, but this was not seen in the present case. Other common locations that carcinosarcoma can spread to include both the cervical and hilar lymph nodes. It is believed that carcinosarcoma spreads using both hematogenous and lymphatic routes owing to its bimodal cell population.^3,9^ However, when taken together the most critical prognostic factors include facial nerve palsy, as seen in this case, the tumor grade as well as the stage, and finally the extent of spread.^10^

The histological morphology of the cells, in the present case, showed both epithelial cells as well as cells of adenomatous character as seen from the mucin staining. Other cases have seen a wide variety of cell origins including elements of the following: chondrosarcoma, fibrosarcoma, leiomyosarcoma, osteosarcoma, and malignant fibrous histiocytoma.^2,3,9,11,12^

Moreover, there remains a great deal of controversy about the actual precursor cells that give rise to carcinosarcoma, which is believed to be a myoepithelial cell.^13,14^ Furthermore, this same myoepithelial cell also is believed to give rise to pleomorphic adenoma of the salivary gland. This, however, remains a point of controversy as other studies have shown that both elements of the carcinosarcoma can arise separately.^3^ Thus, in summation carcinosarcoma is a rare, aggressive, highly invasive, frequently recurrent malignancy that needs to be treated aggressively because of its high rates of recurrence and malignant spread.
